# Atualização dos rastreadores para detecção de eventos adversos a medicamentos em pacientes hematológicos 

**DOI:** 10.1590/0102-311XPT077923

**Published:** 2023-12-22

**Authors:** Íris Pilegi Domingues, Milene Rangel da Costa

**Affiliations:** 1 Universidade Federal do Rio de Janeiro, Rio de Janeiro, Brasil.; 2 Instituto Estadual de Hematologia Arthur de Siqueira Cavalcanti, Rio de Janeiro, Brasil.; 3 Instituto Nacional de Cardiologia, Rio de Janeiro, Brasil.

**Keywords:** Efeitos Colaterais e Reações Adversas Relacionados a Medicamentos, Hematologia, Farmacovigilância, Drug-related Side Effects and Adverse Reactions, Hematology, Pharmacovigilance, Efectos Colaterales y Reacciones Adversas Relacionados con Medicamentos, Hematología, Farmacovigilancia

## Abstract

A utilização de rastreadores para a busca ativa e detecção de eventos adversos a medicamentos (EAM) tem ganhado espaço nos serviços de farmacovigilância. Assim, o objetivo principal do estudo foi propor uma nova lista de rastreadores para ser empregada em um centro especializado em hematologia do Rio de Janeiro, Brasil. A atualização da lista de rastreadores consistiu na revisão da lista atual, com a exclusão e inclusão de rastreadores. Para verificar o desempenho da nova lista de rastreadores, realizou-se um estudo transversal em que os novos rastreadores foram utilizados para investigar a ocorrência de EAM em pacientes atendidos na emergência ou hospitalizados no período de janeiro a março de 2022. Para cada suspeita de EAM identificada, caracterizaram-se o perfil do paciente e as reações adversas a medicamentos (RAM) quanto à causalidade e gravidade. O desempenho dos rastreadores e sua capacidade de captação de EAM foram calculados por meio dos indicadores: frequência do rastreador por 100 prontuários, frequência de EAM por 100 prontuários e valor preditivo positivo (VPP). Para avaliar o desempenho global da nova lista proposta, calculou-se o VPP. Foram identificadas 374 prescrições de rastreadores em 186 prontuários. Os mais eficientes na detecção de possíveis EAM foram: lidocaína, loperamida, bisacodil, filgrastim e clister de glicerina. O VPP global da nova lista sugerida foi 48% contra 10% da lista anterior. Este estudo demonstrou a importância de uma lista de rastreadores atualizada para o monitoramento dos EAM e o aprimoramento da assistência prestada.

## Abstract (en)

The use of triggers for the active search and detection of adverse drug events (ADEs) has been gaining ground within pharmacovigilance services. Thus, the main objective of the study was to propose a new list of triggers to be used in a center specialized in hematology in Rio de Janeiro, Brazil. The update of the list of triggers consisted of revising the current list, with the exclusion and inclusion of new triggers. To verify the performance of the new list of triggers, a cross-sectional study was conducted in which the new triggers were used to investigate the occurrence of ADEs in patients attended in the emergency unit or hospitalized from January to March 2022. For each suspected ADEs, the patient’s profile and adverse drug reactions (ADRs) were characterized regarding causality and severity. The performance of the triggers and their ability to capture ADEs were estimated using the following indicators: frequency of the trigger per 100 medical records, frequency of ADEs per 100 records, and positive predictive value (PPV). To evaluate the overall performance of the proposed new list, the PPV was estimated. A total of 374 prescriptions for triggers were identified in 186 medical records. The most efficient in the detection of possible ADEs were: lidocaine, loperamide, bisacodyl, filgrastim and glycerin clyster. The overall PPV of the new suggested list was 48% versus 10% of the previous list. This study demonstrated the importance of an updated list of triggers for the monitoring of ADEs and improvement of the care provided.

## Resumen

El uso de rastreadores en la búsqueda y detección activa de eventos adversos de medicamentos (EAM) viene ganando espacio dentro de los servicios de farmacovigilancia. Por lo tanto, el objetivo principal de este estudio fue proponer una nueva lista de rastreadores que se utilizarán en un centro de hematología especializado en Río de Janeiro, Brasil. La actualización de la lista de rastreadores consistió en la revisión de la lista actual, con la exclusión e inclusión de rastreadores. Para verificar el desempeño de la nueva lista de rastreadores, se realizó un estudio transversal en el que se utilizaron los nuevos rastreadores para investigar la aparición de EAM en pacientes atendidos en urgencias u hospitalizados en el periodo de enero a marzo de 2022. Para cada sospecha de EAM identificados, el perfil del paciente y las reacciones adversas a medicamentos (RAM) se caracterizaron por su causalidad y gravedad. El desempeño de los rastreadores y su capacidad para capturar EAM se calcularon mediante los indicadores: frecuencia del rastreador por cada 100 registros médicos, frecuencia de EAM por cada 100 registros médicos y valor predictivo positivo (VPP). Para evaluar el desempeño general de la nueva lista propuesta, se calculó el VPP. Se identificaron 374 recetas de rastreadores en 186 registros médicos. Los más eficientes en la detección de posibles EAM fueron lidocaína, loperamida, bisacodilo, filgrastim y enema de glicerina. El VPP general de la nueva lista sugerida fue del 48% frente al 10% de la lista anterior. Este estudio demostró la importancia de una lista actualizada de rastreadores para monitorear EAM y mejorar la atención brindada.

## Introdução

Os eventos adversos a medicamentos (EAM) são definidos pela Organização Mundial da Saúde (OMS) ^1^ (p. 3) como “*qualquer ocorrência médica indesejável que pode ocorrer durante o tratamento com um medicamento, sem necessariamente possuir uma relação causal com o tratamento*”, sendo considerados uma das principais causas de hospitalização e morbidade no mundo ^2^^,^^3^. Em especial, estima-se que os eventos adversos causados por medicamentos, denominados reações adversas a medicamentos, estão associados a cerca de 6,3% das internações em países em desenvolvimento ^4^. Além disso, a proporção de mortes decorrentes de hospitalizações pode alcançar 1,8% em países como Estados Unidos, Espanha, Brasil e África do Sul ^4^^,^^5^. A morbimortalidade associada aos EAM também contribui para o aumento dos custos de saúde ^2^. Segundo Miguel et al. ^6^, os eventos adversos causados por medicamentos induzem custos hospitalares diretos de USD 1,56 bilhão a USD 4 bilhões por ano nos Estados Unidos.

Nesse contexto, as ações de farmacovigilância são fundamentais para garantir a segurança dos medicamentos e determinar a relação de risco-benefício de seu uso ^5^. A farmacovigilância consiste na ciência e nas atividades relativas à identificação, avaliação, compreensão e prevenção de efeitos adversos ou quaisquer problemas relacionados ao uso de medicamentos ^1^. Entre os métodos empregados em farmacovigilância, a notificação espontânea é recomendada pela OMS para ser adotada pelos sistemas nacionais de monitoramento. No entanto, uma das principais limitações desse método é a subnotificação ^7^. Por isso, o emprego de procedimentos de busca ativa, como é o caso do uso de rastreadores, tem ganhado notoriedade na prática dos serviços de farmacovigilância ^8^.

O rastreador é considerado uma pista ou um indício de que possa ter ocorrido um EAM. Assim, quando um medicamento classificado como rastreador é prescrito, as circunstâncias de sua indicação podem ser investigadas por meio das informações contidas no prontuário médico e demais documentos do paciente, de modo a identificar a ocorrência de um possível EAM ^9^. Os pacientes onco-hematológicos constituem uma população especialmente suscetível à ocorrência de EAM, devido à complexidade da sua doença de base e à toxicidade dos tratamentos aos quais são submetidos ^10^^,^^11^. Portanto, a pronta verificação de suspeitas de EAM nessa população é essencial para mitigar possíveis danos causados aos pacientes. Este estudo teve como objetivo aprimorar a metodologia de busca ativa de EAM em um hospital de referência em hematologia por meio da revisão da lista de rastreadores empregada pelo serviço de farmacovigilância da instituição.

## Métodos

Este estudo foi realizado em um centro de referência em hematologia localizado no Município do Rio de Janeiro, Brasil. Trata-se de uma unidade de saúde pública de nível terciário pertencente ao Sistema Único de Saúde (SUS) e especializada no tratamento de doenças hematológicas primárias de alta complexidade. Foram incluídos no estudo pacientes cujo atendimento foi realizado na emergência ou em regime de internação durante o período de 1º de janeiro de 2022 a 31 de março de 2022, sem distinção de sexo e sem limite de idade.

**Figura 1:**
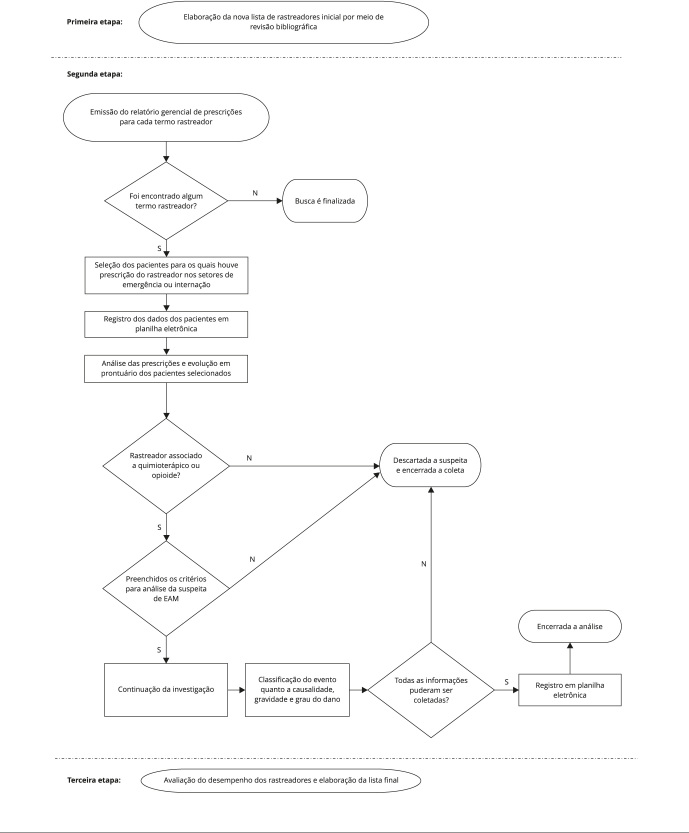
Fluxograma do percurso metodológico.

EAM: eventos adversos a medicamento; N: não; S: sim.

As etapas metodológicas são apresentadas na Figura 1. A primeira consistiu na atualização da lista de rastreadores existente na instituição. Para isso, foi realizada uma revisão bibliográfica a partir de buscas nas bases de dados MEDLINE, via PubMed, e Biblioteca Virtual em Saúde (BVS), utilizando as palavras-chave: “Adverse Drug Reaction Reporting Systems”, “Drug-Related Side Effects”; “Adverse Reactions”; “Pharmacovigilance, trigger tool”; “Patient Safety”. Foram considerados artigos publicados em espanhol, inglês e português, sem limitação de data de publicação.

Para seleção dos rastreadores, foi empregado o critério adotado por Call et al. ^12^, que estabelece os medicamentos rastreadores de acordo com a abrangência de seu uso e a probabilidade de identificar EAM em pacientes hematológicos. De forma complementar, realizou-se o levantamento das principais reações adversas descritas nas bulas dos medicamentos quimioterápicos padronizados na instituição por meio de consulta ao bulário da Agência Nacional de Vigilância Sanitária (Anvisa) ^13^, a fim de mapear o cenário do estudo e nortear a escolha dos rastreadores. Também foram consultados os protocolos de uso de medicamentos da instituição. Apenas rastreadores medicamentosos foram considerados no estudo.

A segunda etapa consistiu na utilização da lista de rastreadores atualizada para detectar possíveis EAM ocorridos durante o período de estudo. O método de detecção de EAM baseou-se nas recomendações do Instituto de Melhoria dos Cuidados de Saúde, dos Estados Unidos (Institute for Healthcare Improvement - IHI) ^9^^,^^14^. De acordo com essa metodologia, é recomendado que 20 prontuários sejam selecionados aleatoriamente a cada mês, os quais são revisados de forma retrospectiva, com foco na identificação dos rastreadores predefinidos pela instituição. O tempo de revisão de cada prontuário não deve ultrapassar 20 minutos. Se um rastreador for identificado em um prontuário, considera-se que seja uma pista de que pode ter ocorrido o EAM e, por isso, deverá ser realizada uma análise mais detalhada do prontuário do paciente, concentrada apenas nas informações que possam ser pertinentes à investigação do evento. Se nenhum evento adverso for encontrado, o revisor deverá seguir a revisão e procurar por outros rastreadores ^14^.

Neste estudo, foi utilizada uma adaptação desse método no que diz respeito à seleção dos prontuários. Foram gerados relatórios gerenciais de prescrição de cada medicamento rastreador por meio do sistema de informações do instituto que continham a relação de todas as prescrições realizadas no período. Esses relatórios também foram fonte de dados sobre a identidade do paciente, data da prescrição do rastreador, via de administração, quantidade prescrita, unidade farmacotécnica e posologia. Os dados de todos os pacientes que tiveram prescrições de rastreadores realizadas durante atendimento na emergência ou internação hospitalar foram coletados e registrados em planilha eletrônica.

A investigação da suspeita do EAM foi realizada para todos os pacientes que receberam a prescrição de pelo menos um medicamento rastreador. Esta consistiu na revisão da prescrição e leitura do prontuário do paciente (físico e/ou eletrônico), sendo verificado o motivo do uso do medicamento. Caso fosse concluído que o uso do rastreador evidenciou a ocorrência de EAM, outro formulário era preenchido, no qual eram registrados dados adicionais dos pacientes (idade, diagnóstico da doença de base de acordo com a 10ª revisão da Classificação Internacional de Doenças - CID-10 ^15^ - e sexo). Se durante a investigação não fosse possível determinar a razão para o uso do medicamento rastreador, aquela prescrição era desconsiderada.

Todos os EAM identificados foram categorizados conforme o Critério Comum de Terminologia para Eventos Adversos (CTCAE) ^16^ e classificados de acordo com sua gravidade segundo a classificação proposta pela OMS ^1^. Os medicamentos suspeitos foram definidos segundo a Classificação Anatômica Terapêutica Química (classificação ATC) ^17^. O algoritmo de Naranjo foi utilizado para avaliar a probabilidade de o evento adverso observado ter sido de fato causado pelo medicamento. De acordo com esse instrumento, a causalidade pode ser classificada como definida, provável, possível ou duvidosa ^18^.

A terceira etapa do estudo consistiu em definir a lista de rastreadores final a partir da avaliação do desempenho dos rastreadores inicialmente selecionados para detecção de EAM. O desempenho dos rastreadores e sua capacidade de captação de EAM foram avaliados utilizando-se os indicadores propostos por Giordani et al. ^19^ e Lipitz-Snyderman et al. ^20^: (1) frequência do rastreador por 100 prontuários (número de registros de cada rastreador dividido pelo número total de prontuários avaliados, multiplicado por 100) ^19^; (2) frequência de EAM por 100 prontuários (número de EAM identificados pelo rastreador dividido pelo número total de prontuários avaliados, multiplicado por 100) ^19^; (3) valor preditivo positivo - VPP - (número de vezes que o rastreador levou à identificação de um EAM dividido pelo total de vezes que o rastreador foi identificado, multiplicado por 100) ^20^. O indicador 1 expressa a carga de trabalho a ser incorporada no processo de identificação do EAM ^19^. Já o indicador 2 demonstra a capacidade de cada rastreador em identificar o EAM ^19^. O VPP revela o potencial de cada um dos rastreadores para a identificação do EAM, ou seja, sua sensibilidade ^20^.

A seleção final dos rastreadores foi definida a partir da análise dos indicadores calculados de acordo com os critérios adotados por Giordani et al. ^19^ e Lipitz-Snyderman et al. ^20^. Giordani et al. ^19^ categorizaram os rastreadores em três grupos de acordo com sua sensibilidade: rastreadores com melhor rendimento (ou seja, sensibilidade de 100%); rastreadores com rendimento intermediário (sensibilidade de 30% a 70%); e rastreadores com baixo rendimento (sensibilidade menor que 30%). Lipitz-Snyderman et al. ^20^ excluíram rastreadores que não foram capazes de identificar EAM ou tiveram o VPP menor que 20%.

Finalmente, com o objetivo de comparar o desempenho global da nova lista de rastreadores em relação à anteriormente empregada na instituição, calcularam-se o VPP global de cada lista ^20^ e a razão entre o número de suspeitas de EAM obtido utilizando a nova lista e o número de suspeitas de EAM encontrado pelo serviço utilizando a lista vigente em 2022, no mesmo período. Os dados foram coletados e as suspeitas de EAM foram analisadas pelo mesmo pesquisador farmacêutico para as duas listas. Todas as análises foram executadas com o auxílio do programa Microsoft Office Excel (https://products.office.com/).

A realização deste estudo foi aprovada pelo Comitê de Ética do Instituto Estadual de Hematologia Arthur de Siqueira Cavalcanti (HEMORIO; parecer CAAE nº 35151020.0.0000.5267).

## Resultados

### Lista inicial de rastreadores elaborada a partir da revisão da literatura

No Quadro 1 são apresentados os 15 medicamentos rastreadores inicialmente selecionados para compor a nova lista de rastreadores da instituição, de acordo com os resultados da revisão bibliográfica. Nele, também são apresentados os EAM associados ao uso de cada rastreador e os critérios para análise das suspeitas de EAM. Alguns medicamentos tipicamente utilizados como rastreadores foram excluídos da lista pelos motivos apresentados a seguir.

**Quadro 1:** Medicamentos rastreadores inicialmente selecionados para compor a nova lista de rastreadores da instituição, eventos adversos associados e critérios de avaliação.

TERMO RASTREADOR	EVENTO ADVERSO ASSOCIADO	CRITÉRIO PARA ANÁLISE DA SUSPEITA DO EAM
Todos	-	Uso prévio ou regular de medicamento quimioterápico.
Alfaepoetina ^3^^,^^31^^,^^32^	Suspeita de anemia ou piora da anemia induzida por quimioterapia.	Exame de hemoglobina < 10g/dL. Relato em prontuário de anemia ou piora da anemia.
Amitriptilina ^32^	Suspeita de parestesia induzida por quimioterápico.	Relato em prontuário de parestesia, neuropatia, neuropatia periférica e/ou formigamento em pés e mãos.
Bisacodil ^14^^,^^32^	Suspeita de constipação induzida por opioides e/ou quimioterápicos.	Uso prévio ou regular de medicamento opioide. Relato em prontuário de constipação ou fecaloma.
Clister de glicerina ^14^^,^^32^	Suspeita de constipação induzida por opioides e alguns quimioterápicos.	Uso prévio ou regular de medicamento opioide. Relato em prontuário de constipação ou fecaloma.
Cloreto de potássio 6% (xarope) ^20^^,^^32^	Hipocalemia induzida por quimioterapia.	Exame laboratorial: potássio sérico < 3mM.
Dexametasona (creme) ^20^^,^^32^	Suspeita de extravasamento no local da infusão do quimioterápico.	Relato em prontuário de extravasamento.
Enoxaparina ^20^^,^^33^	Suspeita de evento tromboembólico induzido por quimioterápico.	Prescrição na posologia de 12/12 horas. Relato em prontuário de embolismo pulmonar, trombose, trombose venosa profunda e infarto.
Filgrastim ^3^^,^^14^^,^^31^	Suspeita de neutropenia ou neutropenia febril induzida por quimioterapia.	Exame laboratorial de neutrófilos absolutos < 1.500/mm^3^. Relato em prontuário de neutropenia ou neutropenia febril.
Gabapentina ^32^	Suspeita de parestesia induzida por quimioterápico.	Relato em prontuário de parestesia, neuropatia, neuropatia periférica e/ou formigamento em pés e mãos.
Lidocaína gel ^20^^,^^34^^,^^35^	Suspeita de mucosite oral.	Prescrição via uso bucal. Relato em prontuário de mucosite, estomatite, odinofagia.
Loperamida ^9^^,^^32^	Diarreia induzida por quimioterapia.	Relato em prontuário de diarreia.
Manitol ^14^	Suspeita de constipação induzida por opioides ou quimioterápicos.	Prescrição via oral. Uso prévio ou regular de medicamento opioide. Relato em prontuário de constipação ou fecaloma.
Poliestirenossulfonato de cálcio ^9^^,^^12^^,^^20^	Suspeita de hipercalemia induzida por quimioterápico.	Exame laboratorial: potássio sérico > 6mM.
Rasburicase ^36^	Suspeita de síndrome de lise tumoral (SLT) induzida por quimioterápico.	Exame laboratorial: ácido úrico > 7,5mg/dL. Relato em prontuário de SLT.
Rivaroxabana ^20^^,33^	Suspeita de evento tromboembólico induzido por quimioterápico.	Relato em prontuário de embolismo pulmonar, trombose, trombose venosa profunda e infarto.

EAM: eventos adversos a medicamento.

Os antieméticos, rastreadores que frequentemente indicam náuseas e vômitos ^21^, foram excluídos porque sua prescrição é preconizada pela instituição em praticamente todos os protocolos de profilaxia de náuseas causadas pela quimioterapia, o que poderia gerar altas taxas de falsos positivos. Os antialérgicos e corticoides também foram descartados, pois ambos são utilizados previamente às transfusões sanguíneas de acordo com os protocolos do instituto. A heparina, apesar de ser um anticoagulante, não foi incluída, uma vez que é utilizada preferencialmente no instituto para ativação e desativação de cateter e em hemodiálise. Os eletrólitos injetáveis, por sua vez, foram descartados, pois algumas vezes são prescritos no campo de observação das soluções de grande volume, como soro fisiológico, o que dificulta sua identificação. Medicamentos que podem induzir hiperglicemia (glicemia > 8,9mM) também foram desconsiderados porque os registros diários da glicemia capilar são realizados pela enfermagem em formulário específico, que nem sempre está prontamente disponível.

### Perfil dos pacientes envolvidos nas suspeitas de eventos adversos a medicamentos

Foram identificadas suspeitas de EAM em um total de 67 pacientes, incluindo adultos, crianças e adolescentes. A maioria dos pacientes tinham mais de 60 anos (22,4%), seguidos daqueles com idades entre 31 e 40 anos (16,4%). Crianças e adolescentes (idade menor ou igual a 18 anos) representaram 19,4% do total. A maioria das suspeitas de EAM foram detectadas em pacientes do sexo masculino (53,7%). Os diagnósticos principais mais frequentes foram leucemia linfoblástica aguda, com 20,9% (14), seguida por leucemia mieloide aguda, com 19,4% (13), e transtornos falciformes, com 16,4% (11).

### Caracterização das suspeitas de eventos adversos a medicamentos

Um total de 374 prescrições de rastreadores foi identificado em 186 prontuários. A média do número de rastreadores por prontuário foi de 2,01 (desvio padrão médio = 1,81), dos quais 58,6% (109) tinham apenas um rastreador e 33,87% (63) entre dois e quatro rastreadores. Após análise dos prontuários, concluiu-se que, do total de prescrições, 98 (26,2%) eram relacionadas a possíveis EAM. Após a retirada das duplicidades, já que oito suspeitas foram identificadas por dois rastreadores ao mesmo tempo, concluiu-se que a lista de rastreadores proposta resultou na detecção de 90 suspeitas de EAM, envolvendo 67 pacientes, o que corresponde a uma incidência de 36 pacientes com suspeitas de EAM a cada 100 pacientes com prescrição de rastreadores (67/186).

Entre os EAM identificados, 55,56% (50) foram distúrbios do sangue e do sistema linfático e 38,89% (35) distúrbios gastrointestinais. Os EAM mais frequentes foram neutropenia febril (36,67%) e constipação (35,56%), conforme mostra a Tabela 1.

**Tabela 1:** Distribuição percentual das suspeitas de eventos adversos a medicamentos (EAM).

EAM	Casos	%
Neutropenia febril	33	36,67
Constipação	32	35,56
Neutropenia	12	13,33
Piora da anemia	4	4,44
Diarreia	2	2,22
Parestesia	2	2,22
Anemia	1	1,11
Evento tromboembólico (trombose)	1	1,11
Hipocalemia	1	1,11
Mucosite	1	1,11
Síndrome de lise tumoral	1	1,11
Total de EAM identificados	90	100,00

Considerando o segundo nível da classificação ATC, que se refere ao subgrupo terapêutico, dez classes de medicamentos estiveram envolvidas nas suspeitas de EAM, totalizando 41 medicamentos suspeitos. Os agentes antineoplásicos (L01) foram os mais frequentes, correspondendo a 60,98% (25) do total de medicamentos, principalmente a citarabina, a ciclofosfamida e a fludarabina. Outros medicamentos envolvidos nas suspeitas de EAM pertenciam aos subgrupos terapêuticos dos psicoanalépticos (N06, 7,32%), antibacterianos para uso sistêmico (J01, 7,32%) e analgésicos (N02, 4,88%).

Entre os EAM detectados com a nova lista de rastreadores, 163 (84%) foram classificados como eventos possivelmente causados pelo medicamento e 30 (16%) foram definidos como provavelmente causados pelo medicamento, de acordo com o algoritmo de Naranjo. Cabe esclarecer que mais de um medicamento suspeito pode estar envolvido na mesma suspeita de EAM e, por isso, a quantidade de análises realizadas com algoritmo de Naranjo (193) foi superior ao número de EAM detectados no estudo (90).

Os eventos graves totalizaram 87,78% (79), sendo os demais classificados como não graves. A citarabina foi o medicamento mais frequentemente envolvido em EAM graves que causaram hospitalização ou prolongamento da hospitalização, correspondendo a 17,24% dos EAM dessa classe. A fludarabina foi o medicamento mais frequentemente relacionado a EAM graves por outro efeito clinicamente significativo (13,76%). Morfina, tramadol e omeprazol foram os medicamentos mais recorrentemente associados a EAM não graves, sendo responsáveis, em conjunto, por cerca de 69% desses EAM.

### Desempenho dos rastreadores selecionados

A Tabela 2 apresenta os resultados da avaliação de desempenho dos rastreadores da lista inicialmente proposta. Os rastreadores que exibem maior frequência de prescrição por 100 prontuários foram filgrastim (46,24 por 100 prontuários), amitriptilina (28,49 por 100 prontuários) e clister de glicerina (20,43 por 100 prontuários). Dos 15 rastreadores inicialmente selecionados, 12 levaram à identificação de possíveis EAM. Os rastreadores com maior número de EAM identificados por 100 prontuários foram filgrastim (24,19 por 100 prontuários), clister de glicerina (10,22 por 100 prontuários) e bisacodil (10,22 por 100 prontuários).

**Tabela 2:** Avaliação do desempenho dos rastreadores de acordo com os indicadores selecionados.

Termo rastreador	Frequência do rastreador por 100 prontuários	Frequência do EAM por 100 prontuários	VPP
Lidocaína gel	0,54	0,54	100,00
Loperamida	1,61	1,08	66,67
Bisacodil	18,82	10,22	54,29
Filgrastim	46,24	24,19	52,33
Clister de glicerina	20,43	10,22	50,00
Alfaepoetina	11,29	2,69	23,81
Rasburicase	3,23	0,54	16,67
Gabapentina	8,60	1,08	12,50
Rivaroxabana	13,44	0,54	4,00
Cloreto de potássio (xarope)	15,05	0,54	3,57
Enoxaparina	15,05	0,54	3,57
Amitriptilina	28,49	0,54	1,89
Dexametasona creme	13,44	0,00	0,00
Manitol	0,54	0,00	0,00
Poliestireno de cálcio	4,30	0,00	0,00
Global	201,08	52,69	26,20

EAM: eventos adversos a medicamento; VPP: valor preditivo positivo.

Nota: total de prontuários avaliados = 186.

Os VPP calculados para os rastreadores variaram entre 0 e 100 (Tabela 2). A lidocaína gel foi o rastreador de melhor VPP (100%), ou seja, em todas as vezes que foi prescrita, sinalizou um EAM. Nesse caso, o EAM foi a ocorrência de casos graves de mucosite que levaram à hospitalização ou ao prolongamento da hospitalização. Os rastreadores loperamida, bisacodil, filgrastim e clister de glicerina foram rastreadores com VPP intermediário, ou seja, a sensibilidade de captação de EAM foi de 30% a 70% ^20^. Entre os rastreadores com VPP abaixo de 30% incluem-se alfaepoetina (23,81%), rasburicase (16,67%), gabapentina (12,5%), rivaroxabana (4%), cloreto de potássio (xarope) (3,57%), enoxaparina sódica (3,57%) e amitriptilina (1,89%). Já os medicamentos dexametasona, manitol e poliestirenossulfonato de cálcio apresentaram VPP nulo, ou seja, não identificaram nenhum EAM. O VPP global da nova lista de rastreadores foi de 26,2%.

Todos os 12 rastreadores que resultaram na identificação de suspeitas de EAM sinalizaram ao menos um EAM grave. No caso do rastreador filgrastim, todos foram graves, sendo que 52,78% (19) demandaram hospitalização ou prolongaram a internação.

### Desempenho dos rastreadores da lista utilizada pela instituição

A lista de rastreadores atualmente usada pelo serviço de farmacovigilância da instituição contém apenas quatro medicamentos rastreadores que são considerados medicamentos antídotos segundo o IHI, a saber: flumazenil, cloridrato de naloxona, protamina e vitamina K ^14^^,^^22^. No período entre 1º de janeiro de 2022 e 31 de março de 2022, um total de dez prescrições de rastreadores foi identificado pelo serviço, referente a nove prontuários. A média do número de rastreadores por prontuário foi de 1,11 (desvio padrão médio = 0,31), dos quais 88,89% (8) tinham apenas um rastreador e 11,11% (1) apresentavam dois rastreadores. Após a investigação, concluiu-se que, do total de prescrições de rastreadores identificadas no período, apenas o flumazenil de fato detectou uma suspeita de EAM em um único prontuário, resultando em um VPP igual a 16,67%. O medicamento suspeito nesse EAM foi o midazolam. O cloridrato de naloxona foi identificado uma única vez, porém foi prescrito para a reposição do carro de urgência e emergência, sem associação ao uso por paciente. A vitamina K foi detectada em três prescrições, entretanto seu uso estava associado à doença hematológica de base. A protamina não foi prescrita no período do estudo. O VPP global da lista em uso na instituição foi igual a 10% e a razão entre o número de suspeitas de EAM obtido utilizando a nova lista e o número de suspeitas de EAM encontrado pelo serviço utilizando a lista vigente no mesmo período foi igual a 90 (90/1).

### Lista de rastreadores após a análise de desempenho

Após a análise de desempenho dos rastreadores, e considerando os critérios propostos por Lipitz-Snyderman et al. ^20^, os rastreadores que não foram capazes de identificar EAM ou apresentaram VPP menor que 20% seriam excluídos, resultando em uma lista final contendo os rastreadores apresentados no Quadro 2, com exceção do flumazenil e do cloridrato de naloxona, o que elevaria o VPP global da nova lista para 48%. No entanto, tendo em vista as conclusões de Call et al. ^12^ - de que medicamentos antídotos, como flumazenil e cloridrato de naloxona, podem ser relevantes como rastreadores -, o fato de que estes são também recomendados pela ferramenta do IHI ^9^^,^^14^ e que medicamentos benzodiazepínicos e opioides são frequentemente prescritos na instituição, decidiu-se por incluí-los na lista final.

**Quadro 2:** Lista de rastreadores medicamentosos proposta após análise de desempenho dos rastreadores.

RASTREADOR
Alfaepoetina Bisacodil Clister de glicerina Filgrastim Flumazenil Lidocaína gel Loperamida Cloridrato de naloxona

## Discussão

Este estudo resultou na proposta de uma nova lista de rastreadores medicamentosos a serem empregados para detecção de EAM em pacientes onco-hematológicos. A nova lista demonstrou sensibilidade significativamente superior à da lista atual da instituição (VPP 48% *versus* 10%) e, consequentemente, maior capacidade de identificação de EAM. A metodologia proposta pelo IHI para detecção de EAM ^14^ foi adaptada com o objetivo de melhor se adequar ao perfil dos pacientes do instituto, às suas configurações de cuidado e aos recursos disponíveis, já que os termos rastreadores propostos pelo IHI são genéricos. Tal estratégia se mostrou alinhada com a de outros pesquisadores, conforme descrito na revisão sistemática e metanálise realizada por Eggenschwiler et al. ^23^. A identificação preliminar dos rastreadores a partir do sistema de informações do instituto tem a vantagem de reduzir o tempo necessário para as análises. Além disso, permitiu que todos os rastreadores prescritos no período fossem analisados, e não apenas aqueles contidos em uma amostra de vinte prontuários ao mês, como recomenda o método do IHI ^9^^,^^14^, evitando-se, assim, um possível viés de seleção. Ainda, todas as etapas do estudo, inclusive aquelas relacionadas à lista previamente usada pelo serviço, foram realizadas pelo mesmo profissional farmacêutico, com experiência na área de farmacovigilância e hematologia. Embora o sistema informatizado tenha otimizado o tempo investido nas análises, o prontuário eletrônico ainda não é usado por toda a equipe multiprofissional, o que demandou que os prontuários físicos também fossem consultados, levando ao aumento do tempo necessário para as investigações. Como observado por Giordani et al. ^19^, outras desvantagens de registros manuais são a legibilidade prejudicada e o uso de abreviaturas.

Os rastreadores filgrastim, clister de glicerina e bisacodil foram capazes de identificar maior número de eventos, o que já era esperado, visto que foram os rastreadores mais prescritos. Esses rastreadores também foram os que identificaram maior número de EAM graves, confirmando, assim, a importância de sua seleção para monitoramento dos EAM da instituição. Gerber et al. ^11^ investigaram o uso de rastreadores para detecção de EAM em pacientes com câncer hematológico e tumor sólido hospitalizados em três hospitais da Suíça. Este estudo usou alguns rastreadores semelhantes ou monitorou eventos adversos parecidos por meio de rastreadores diferentes. Enquanto Gerber et al. ^11^ utilizaram como termos rastreadores os próprios EAM, como mucosite, diarreia, constipação, extravasamento, síndrome de lise tumoral, anticoagulação e hipercalemia, este artigo avaliou esses mesmos EAM, porém por meio dos respectivos termos rastreadores, como lidocaína gel (via uso bucal), loperamida, bisacodil, clister de glicerina e manitol (uso oral). A comparação dos resultados dos estudos pode ter sido prejudicada por diferenças no perfil dos pacientes, já que no estudo de Gerber et al. ^11^ a proporção de pacientes com câncer hematológico era menor do que a proporção de pacientes com tumores sólidos.

Lipitz-Snyderman et al. ^20^ também avaliaram a performance de rastreadores na identificação de EAM em oncologia. Embora o perfil dos pacientes seja diferente, já que incluiu casos de câncer de mama, colorretal e de pulmão, alguns termos rastreadores selecionados se assemelham aos deste estudo. Assim como Gerber et al. ^11^, os autores também utilizaram o próprio EAM como termo rastreador, a exemplo da neutropenia febril, para o qual encontrou o VPP de melhor rendimento (100%). Assim como nesta pesquisa, Lipitz-Snyderman et al. ^20^ encontraram um alto VPP para anestésicos bucais, como a lidocaína.

Os rastreadores vitamina K e cloridrato de naloxona incluídos na lista de rastreadores original da instituição também foram utilizados por Gerber et al. ^11^. De maneira similar a este estudo, os autores também encontraram VPP de baixo desempenho ou nulo para esses rastreadores. De acordo com Giordani et al. ^19^, a não utilização do cloridrato de naloxona pode ser justificada pela dificuldade de diagnosticar intoxicação por opioides ou, contrariamente, em razão de tanto o diagnóstico como a intervenção serem tempestivos, prescindindo do uso de antagonistas.

Call et al. ^12^ também utilizaram rastreadores para monitorar EAM em um hospital pediátrico especializado em oncologia, hematologia e outras doenças graves. Entre os termos rastreadores usados por eles, estavam contemplados também os quatro medicamentos antídotos contidos na lista original do serviço de farmacovigilância. Similarmente a esta análise, os pesquisadores encontraram VPP nulo para protamina e vitamina K. Esse grupo sugeriu melhorar a sensibilidade do rastreador vitamina K, restringindo-o ao uso prévio ou atual da varfarina.

De forma geral, os rastreadores antídotos apresentaram um VPP baixo ou nulo. De acordo com Gerber et al. ^11^, esses achados poderiam ser justificados pelo baixo desempenho do rastreador e/ou pelas baixas taxas de eventos associados, sendo necessárias mais pesquisas para definir os rastreadores de melhor desempenho para determinada população. Call et al. ^12^ consideraram que os rastreadores que apresentam baixa frequência de prescrição deveriam ter a análise prolongada, como é o caso dos antídotos, já que podem ser úteis para detecção de EAM. Com isso, optou-se por incluir os antídotos flumazenil e cloridrato de naloxona na lista de rastreadores proposta. A partir da utilização dessa lista por períodos maiores na instituição, sua inclusão poderá, então, ser reavaliada.

O fato de o VPP global encontrado para a lista proposta ser maior que o da lista originalmente empregada pelo serviço de farmacovigilância do instituto pode ser devido à utilização de termos rastreadores mais específicos, evidenciando-se que a utilização apenas de medicamentos rastreadores antídotos propostos pelo IHI ^9^^,^^14^ não é adequada ao monitoramento de EAM na instituição. Isso corrobora a posição de Hébert et al. ^24^ de que a ferramenta do IHI é genérica e que não incorpora as especificidades da área de oncologia. Call et al. ^12^ utilizaram em seu estudo uma lista de rastreadores parecida com a lista original da instituição e encontraram baixo VPP global, concluindo que para que a detecção fosse mais eficiente, os rastreadores deveriam ser revisados a fim de melhor refletir a população de pacientes pediátricos hematológicos e oncológicos.

Os eventos adversos a medicamentos representam uma constante preocupação nos sistemas de saúde ^25^. Podem resultar em sequelas permanentes nos indivíduos e custos para as instituições de saúde e para a sociedade, em um contexto atual em que os recursos disponíveis para saúde são limitados ^26^. Como consequência dos EAM, podem-se citar a redução do sucesso do tratamento e a geração de possíveis novos problemas de saúde, além do maior consumo de recursos devido ao aumento do tempo de internação, aos atendimentos de emergência e à maior taxa de morbidade e mortalidade ^27^^,^^28^^,^^29^. As doenças hematológicas, em especial, apresentam um alto potencial de dano sistêmico. São doenças crônicas que requerem tratamentos contínuos, o que torna os pacientes mais suscetíveis a diferentes complicações, como infecções e hemorragias, causando importantes reflexos nos campos social e psicológico ^30^. A quimioterapia, modalidade comumente empregada no tratamento desses pacientes, tem um potencial tóxico e torna o paciente especialmente suscetível à ocorrência de eventos adversos. Nesse contexto, o desenvolvimento de ações de farmacovigilância com o objetivo de monitorar a ocorrência de eventos adversos a medicamentos é fundamental para garantir a segurança do paciente hematológico e reduzir os custos da assistência à saúde para esses pacientes.

## Conclusão

Os resultados deste estudo demonstraram que a nova lista de rastreadores sugerida foi capaz de detectar maior número de EAM do que aquela previamente usada pelo serviço. Além disso, é mais específica para o perfil dos pacientes da instituição, o que contribui para o melhor monitoramento da segurança e assistência prestada. Estudos futuros com maior tamanho amostral e maior tempo de observação, que permitam uma análise mais aprofundada sobre o desempenho dos rastreadores, sobretudo dos antídotos flumazenil e cloridrato de naloxona, poderiam proporcionar um melhor entendimento sobre a diferença de desempenho entre a lista atual da instituição e a nova lista proposta. Além disso, a inclusão de rastreadores laboratoriais também merece ser estudada, pois poderia contribuir para o aumento da capacidade de detecção de EAM.
